# VEA-net: vascular enhancement attention with dual-backbone multi-task learning for comprehensive ROP management across multi-center datasets

**DOI:** 10.3389/fcell.2026.1865503

**Published:** 2026-07-24

**Authors:** Yuan Chen, Jiajun Wan, Tian Zhang, Zhijiang Wan, Jin Hong, Youpeng Jin

**Affiliations:** 1 Department of Pediatrics, Shandong Provincial Hospital Affiliated to Shandong First Medical University, Jinan, Shandong, China; 2 College for Excellent Engineers, Nanchang University, Nanchang, Jiangxi, China; 3 School of Artificial Intelligence, Nanchang University, Nanchang, Jiangxi, China

**Keywords:** computer-aided diagnosis, convolutional neural networks, deep learning, domain adaptation, multi-task learning, retinopathy of prematurity, treatment decision support, vascular enhancement

## Abstract

**Background:**

Retinopathy of Prematurity (ROP) is a vasoproliferative retinal disorder and a major cause of childhood blindness worldwide. Clinical ROP management involves three correlated tasks: Plus disease detection, stage classification, and treatment decision-making. Although deep learning (DL) has shown promise for automated ROP management, several challenges remain: (1) retinal vascular morphology is central to Plus disease assessment, but DL-based vascular feature modeling is not always explicitly integrated into unified end-to-end multi-task frameworks; (2) Plus disease detection, stage classification, and treatment decision-making are clinically related, yet they are often modeled as separate tasks; and (3) model robustness may be affected by class imbalance and domain shifts across heterogeneous datasets.

**Methods:**

We propose VEA-Net, a novel dual-backbone multi-task learning framework that addresses these challenges through three core components: (1) We introduce the Vascular Enhancement Attention (VEA) module, which explicitly models and enhances vascular features through multi-scale convolutions, directional selective filtering, and dual attention mechanisms. (2) We develop a hierarchical multi-task learning architecture that jointly optimizes Plus detection, stage classification, and treatment decision-making while leveraging task correlations through hierarchical consistency losses. (3) We implement a dual-domain adaptation strategy combining Domain-Adversarial Neural Networks (DANN) with Maximum Mean Discrepancy (MMD) to learn domain-invariant representations across heterogeneous data sources.

**Results:**

We validate VEA-Net on three public ROP datasets (FARFUM-ROP, Ostrava, and A-Fundus), comprising 8,636 retinal images from different geographic regions and imaging settings. Using subject-level 10-fold cross-validation with Group K-Fold splitting, our model achieves test AUROC of 91.76% ± 4.16% for Plus detection, 88.26% ± 4.61% for stage classification, and 66.81% ± 15.87% for treatment decision-making.

**Conclusion:**

VEA-Net provides a unified framework for image-based ROP management by integrating vascular enhancement, hierarchical multi-task learning, and domain adaptation. The model supports Plus detection, stage classification, and auxiliary treatment decision support within a single architecture. The results indicate VEA-Net can learn robust representations across heterogeneous public datasets, and potential for improving ROP management efficiency.

## 1 Introduction

Retinopathy of Prematurity (ROP) is a vasoproliferative retinal disorder primarily affecting premature infants born before 31 weeks of gestation or with a birth weight less than 1,500 g. As a leading cause of childhood blindness worldwide, ROP affects a substantial number of infants globally, with variations in epidemiology driven by disparities in neonatal care and a notably higher prevalence in certain developing regions (Wang et al., 2024; Blazon et al., 2024). The disease progresses through distinct stages characterized by abnormal retinal vascular development, ranging from mild peripheral vascular changes to severe retinal detachment. Effective ROP management requires three clinically relevant tasks: (1) Plus disease detection; (2) stage classification; and (3) treatment decision-making, which determines whether immediate intervention, close monitoring, or routine follow-up is required (Chiang et al., 2021). These tasks are clinically interdependent, because Plus disease status and disease stage jointly inform treatment-related decisions. Accurate and timely ROP assessment is therefore important for preventing irreversible vision loss and improving outcomes in affected premature infants.

Traditional approaches to ROP diagnosis rely heavily on manual examination of retinal fundus images by experienced ophthalmologists, a process that can be time-consuming and subject to inter-examiner variability. More broadly, recent deep learning studies across diverse data-intensive domains have demonstrated the ability of neural networks to learn complex representations from heterogeneous data (Turab et al., 2026; Jabbar et al., 2025; Ullah et al., 2025; Ullah et al., 2024; Rehman et al., 2025). In medical imaging, this representational capacity has supported image-based feature learning, disease classification, and computer-aided diagnosis (Arya et al., 2023; Khvostikov et al., 2018; Zhang et al., 2024; Cazacu et al., 2019). This momentum has naturally extended to ophthalmology, where DL can support image-based disease classification, screening, and clinical decision support across ocular diseases (Xu and Yang, 2023). Motivated by these advances, many studies have introduced DL methods into ROP diagnosis, achieving encouraging performance in Plus disease detection and stage classification (Brown et al., 2018; Chen et al., 2021; Huang et al., 2020).

However, DL methods for ROP clinical management continue to face several limitations that may affect their applicability in heterogeneous clinical settings. Specifically, although previous ROP studies have achieved encouraging performance in Plus disease detection (Brown et al., 2018; Chen et al., 2021; Li and Liu, 2022; Wang et al., 2021; Wu et al., 2022), severity assessment, and multi-component screening systems, vascular morphology is not always explicitly integrated into unified end-to-end multi-task frameworks. This is clinically relevant because Plus disease is defined primarily by posterior pole vascular dilation and tortuosity (Chiang et al., 2021). Furthermore, neonatal retinal vascular pathology is closely related to abnormal vascular development and oxygen-related vascular changes (Xu et al., 2025). Standard convolutional models may learn discriminative global image patterns, but they do not necessarily provide dedicated mechanisms for enhancing vessel caliber, tortuosity, and related morphological patterns that are central to ROP assessment. In addition, many existing automated ROP systems focus on individual clinical endpoints, such as Plus disease detection, stage classification, image quality assessment, or treatment-requiring ROP screening, rather than jointly modeling the full diagnostic workflow (Brown et al., 2018; Huang et al., 2020; Wang et al., 2021; Shoeibi et al., 2026). In clinical practice, however, Plus disease status, disease stage, and treatment-related decisions are interdependent. A unified multi-task framework may therefore help capture shared representations across related tasks while preserving task-specific outputs. Moreover, variations in fundus image quality and acquisition conditions may affect automated retinal image analysis. Retinal image enhancement studies have shown that improving vessel and structural visibility can support more reliable computer-aided interpretation under suboptimal imaging conditions (Wan et al., 2022). Class imbalance and multi-center heterogeneity remain important obstacles for ROP model evaluation. Differences in imaging devices, acquisition protocols, image quality, and patient populations may introduce domain shifts that reduce performance when a model is applied outside the distribution on which it was optimized (Guan and Liu, 2021; Mahapatra et al., 2024; Liu et al., 2026). These issues motivate the development of models that explicitly consider vascular morphology, task relationships, image-level robustness, and retrospective multi-dataset robustness, while still requiring prospective validation in completely unseen clinical centers.

To address these challenges, we propose VEA-Net (Vascular Enhancement Attention Network), a dual-backbone multi-task learning framework for comprehensive image-based ROP analysis. Our approach integrates three core components: (1) a Vascular Enhancement Attention (VEA) module that models and enhances vascular features through multi-scale convolutions, directional selective filtering, and dual attention mechanisms; (2) a hierarchical multi-task learning architecture that jointly optimizes Plus disease detection, stage classification, and image-based auxiliary treatment decision support while leveraging task correlations through hierarchical consistency losses; and (3) a dual-domain adaptation strategy combining Domain-Adversarial Neural Networks (DANN) with Maximum Mean Discrepancy (MMD) to learn more domain-invariant representations across heterogeneous data sources. We validate our method on three public ROP datasets, including FARFUM-ROP (Akbari et al., 2024), Ostrava (Timkovič et al., 2024), and A-Fundus (Zhao et al., 2024), comprising 8,636 retinal images from different geographic regions and imaging settings.

The main contributions of this work are summarized as follows: (1) We propose the VEA module to enhance retinal vascular features that are relevant to ROP assessment. By combining multi-scale feature extraction, directional selective convolution, and dual attention, VEA is designed to improve the representation of vascular dilation, tortuosity, and related morphological patterns. (2) We develop a hierarchical multi-task learning framework that jointly models Plus disease detection, stage classification, and image-based auxiliary treatment decision support. The framework incorporates hierarchical consistency losses to encourage clinically plausible relationships among the three tasks while preserving task-specific prediction heads. (3) We introduce a dual-domain adaptation strategy to mitigate feature distribution shifts across heterogeneous public datasets. By combining adversarial domain confusion with explicit distribution alignment, our method aims to improve representation robustness across different imaging sources.

The remainder of this paper is organized as follows: Section 2 reviews related work on DL for ROP and domain adaptation; Section 3 details the datasets and methods employed in this study; Section 4 presents experimental results and comparative analysis; Section 5 discusses clinical implications and limitations; Section 6 concludes with future directions.

## 2 Related work

### 2.1 Deep learning for ROP detection and classification

Early automated approaches demonstrated that CNN-based models could detect severe Plus disease from retinal fundus images (Brown et al., 2018). Subsequent studies extended this direction to multi-class staging and quantitative feature extraction. For example, Li et al. developed an automated DCNN-based system for early-stage ROP categorization and geometrical feature extraction of demarcation lines (Li and Liu, 2022). These studies established the feasibility of using DL for image-based ROP grading. Beyond these early efforts, several studies have moved toward more comprehensive screening systems by integrating multiple clinically relevant outputs. Wang et al. proposed an explainable multidimensional DL platform that incorporated multiple classifiers, including image quality, stage, hemorrhage, and Plus disease, to support automated ROP screening (Wang et al., 2021; Wu et al., 2022). Shoeibi et al. further evaluated different CNN architectures, including MobileNet and DenseNet, in telemedicine-oriented settings and reported high diagnostic sensitivity for treatment-requiring ROP cases (Shoeibi et al., 2026). These studies indicate that multi-component ROP analysis is clinically meaningful, although many existing systems still treat individual outputs as separate modules rather than explicitly optimizing Plus disease detection, stage classification, and treatment-related decision support within a unified multi-task framework.

Recent methods have increasingly emphasized lesion localization, vascular structure modeling, and feature representations beyond standard spatial convolution. Qi et al. proposed MSLI-Net, which uses multi-segment localization and multi-scale interaction to identify retinal lesions across different receptive fields (Qi et al., 2025). Cheng et al. introduced WaveNet-SF, a hybrid network that incorporates wavelet transforms in the spatial-frequency domain to model vascular and structural patterns beyond standard spatial convolutions (Cheng et al., 2025). In parallel, retinal image enhancement methods have been developed to improve the visibility of fundus structures under suboptimal imaging conditions, which is relevant for ROP images acquired across heterogeneous devices and centers (Wan et al., 2022). These studies highlight the importance of explicitly considering vascular morphology, lesion localization, and image quality in retinal image analysis. More recently, retinal and medical image learning studies have introduced broader methodological advances that are relevant to ROP but remain underexplored in pediatric ROP datasets. Retinal foundation models trained with large-scale self-supervised learning have demonstrated transferable representations across multiple retinal disease detection tasks (Zhou et al., 2023). Complementing these advances, multi-rater medical image learning has emphasized annotation uncertainty and inter-rater variability, showing that expert labels may not always represent a single absolute ground truth (Wu et al., 2024). These directions are relevant to ROP because pediatric retinal datasets are often limited in size, imaging conditions vary across centers, and labels such as disease stage and treatment need may involve expert-dependent interpretation.

While the aforementioned methods aim to improve the representation of retinal structures, they either operate as pre-processing steps, replace the backbone entirely, or focus on generic lesion regions without specifically targeting the vascular abnormalities that define Plus disease. In contrast, our VEA module is designed as a lightweight plug-in attention mechanism that explicitly enhances vascular features at the intermediate representation level. By jointly modeling vascular continuity and local texture patterns through a hybrid spatial-channel attention pathway, the VEA module adaptively amplifies diagnostically critical vascular signals (e.g., vessel tortuosity and dilation) while suppressing background fundus artifacts, without imposing additional pre-processing pipelines or requiring architectural overhaul. This targeted vascular enhancement is particularly suited to ROP diagnosis, where subtle vascular changes are the primary discriminative cues for Plus disease and, by extension, stage progression and treatment decisions.

### 2.2 Multi-task learning in medical image analysis

Multi-task learning (MTL) has emerged as a powerful paradigm in medical image analysis by leveraging shared representations across related tasks. In microscopic and histopathological imaging, Pan et al. introduced a cost-sensitive MTL framework to simultaneously address nuclear segmentation and classification while combating imbalanced annotations (Pan et al., 2023). Similarly, Xing et al. proposed a multi-task contrastive learning approach with multi-scale uncertainty estimation to tackle class imbalance in semi-supervised medical image segmentation (Xing et al., 2023). To reduce the dependency on massive manually annotated datasets, Han et al. explored a meta multi-task learning model that achieves robust nuclei segmentation with significantly fewer training samples (Han et al., 2022). In cellular image analysis, Percannella et al. proposed an end-to-end multi-task U-Net model that simultaneously handles cell intensity classification, specimen segmentation, and pattern recognition (Percannella et al., 2025).

The challenge of extracting intricate features from noisy, complex backgrounds is equally critical in *in vivo* medical imaging. For instance, in endoscopic ultrasound (EUS) analysis, artificial intelligence has driven a new wave of innovation by overcoming traditional limitations such as high interobserver variability (Liu E. et al., 2021). Advanced DL algorithms have successfully enabled the precision diagnosis of challenging lesions, such as pancreatic ductal adenocarcinoma, achieving high sensitivity and specificity despite the inherently noisy nature of ultrasound images (Prasoppokakorn et al., 2022). Furthermore, the integration of real-time AI guidance in these dynamic environments is proving transformative for intraprocedural clinical outcomes (Mahajan et al., 2025). The proven ability of AI to capture subtle structural abnormalities in such challenging modalities demonstrates its robust, universal feature extraction capabilities. This cross-modality success directly inspires our methodology for ROP analysis, where identifying delicate, tortuous retinal vascular networks against varying fundus backgrounds poses a similarly demanding feature extraction challenge.

Prior work has demonstrated the broad applicability of multi-task learning across diverse medical imaging domains, from histopathology to endoscopy. However, these MTL frameworks typically treat the co-learned tasks as peers with equal status, optimizing them via simple loss summation without explicitly encoding the clinical hierarchies that govern real-world diagnostic workflows. In ROP management, this limitation is particularly consequential: Plus disease detection, stage classification, and treatment decision-making do not constitute independent problems but rather form a cascaded clinical decision chain in which each level naturally constrains and informs the next. Existing multi-component ROP platforms address this gap only partially by assembling several independent classifiers into a single pipeline (Wang et al., 2021), which fails to leverage shared representations across tasks and may produce clinically inconsistent predictions. Our hierarchical multi-task learning framework departs from this paradigm by jointly optimizing all three tasks within a unified architecture while introducing explicit hierarchical consistency losses that penalize clinically implausible prediction combinations (e.g., a high-stage prediction without corresponding Plus indicators, or a treatment recommendation inconsistent with the predicted stage). This design not only improves individual task performance through positive transfer but also ensures that the model’s predictions remain clinically coherent—a property that is difficult to guarantee when tasks are optimized independently or treated as undifferentiated peers.

### 2.3 Domain adaptation for medical imaging

Domain shift remains a critical challenge when deploying models across different institutions or imaging devices. Guan et al. provided a comprehensive survey on how domain adaptation strategies successfully handle distribution shifts in medical image analysis without requiring extensive labeled target data (Guan and Liu, 2021). To maintain high performance across varying clinical environments, Mahapatra et al. proposed an active learning pipeline incorporating feature disentanglement and domain adaptation, effectively mitigating cross-scanner variations (Mahapatra et al., 2024). In multi-modality scenarios, Jiang et al. demonstrated the efficacy of cycle-consistency generative adversarial networks (CycleGANs) in generating hallucinated MRI features from CT scans, successfully regularizing cross-modality domain adaptation for tumor segmentation (Jiang et al., 2018). In the context of ophthalmology, ensuring robust feature extraction across varying fundus camera qualities is paramount. To this end, Liu et al. recently proposed DGSSA, a domain generalization framework that employs structural and stylistic augmentation to ensure robust retinal vessel segmentation across diverse, unseen datasets (Liu et al., 2026). These domain adaptation and generalization strategies are vital for building robust networks like VEA-Net, where the heterogeneity of multi-center ROP fundus cameras necessitates strong feature alignment to maintain clinical reliability. More broadly, recent transfer-learning studies in data-scarce heterogeneous environments have also emphasized the value of feature alignment and domain-adversarial learning for improving model robustness under distribution shift (Mahmood et al., 2025), although such non-medical applications are not directly comparable to ROP image analysis.

Previous studies have largely relied on single-mechanism strategies, such as image-level synthesis via generative models, complex active learning pipelines, or stylistic data augmentation. In contrast, our approach distinguishes itself by employing a dual-domain adaptation strategy directly at the feature level. Specifically, VEA-Net seamlessly integrates DANN with MMD to align the heterogeneous feature distributions across multi-center datasets. Unlike methods that demand target-domain annotations or computationally expensive image-to-image translation, our synergistic approach ensures both global domain confusion and local statistical alignment. This provides a lightweight yet robust solution to handle the severe domain shifts caused by varying ROP imaging devices, thereby maintaining high clinical reliability across all three hierarchical diagnostic tasks simultaneously.

## 3 Materials and methods

### 3.1 Dataset description

We evaluate VEA-Net on three large-scale public ROP datasets collected from different geographic regions and imaging centers. These datasets collectively comprise 8,636 retinal fundus images, providing diverse representation across imaging devices, population characteristics, and disease severity distributions. Specifically, the FARFUM-ROP dataset was collected from the Farabi and Ferdowsi University of Mashhad’s (FARFUM) ROP project in Iran, containing 1,533 retinal images from 68 premature infants captured using RetCam III imaging systems. This dataset provides the most comprehensive clinical annotations, including three-class Plus disease status (e.g., Normal, Pre-Plus, Plus), ICROP stage labels, and treatment recommendations made by experienced pediatric ophthalmologists. Due to its complete annotation coverage across all three tasks, FARFUM-ROP serves as the primary source domain for supervised training. The Ostrava dataset was collected from the University Hospital Ostrava in the Czech Republic and contains 6,004 retinal images from 188 patients captured using three different imaging systems. The original dataset provides binary Plus disease labels (Normal/Plus) and a richer set of diagnosis codes: physiological normal, ROP Stages 0–5 (including Stage 4A and 4B), aggressive posterior ROP, status post ROP, and several non-ROP posterior segment diagnoses. We map these diagnosis codes to the standard ICROP Stage 0–5 scheme (e.g., ROP 4A and 4B both map to Stage 4, aggressive posterior ROP maps to Stage 4, status post ROP maps to Stage 5, and non-ROP diagnoses are excluded from stage supervision). The Ostrava dataset, with its multi-device acquisition and European patient population, is valuable for cross-center validation and domain adaptation experiments. The A-Fundus dataset was collected from the Shenzhen Eye Hospital in China and contains 1,099 retinal fundus images from 483 premature infants, organized into five categories: Normal, Stage 1, Stage 2, Stage 3, and Laser Scars (Zhao et al., 2024). Because ROP conditions are progressive, some infants have multiple follow-up images; 342 out of 483 infants (70.8%) have more than one image. After excluding 2 subjects with incomplete metadata, 1,079 images from 481 infants were included in our experiments. This dataset primarily contributes to the stage classification task. Table 1 summarizes the characteristics of the three datasets, including label availability and per-class sample counts.

**TABLE 1:** Summary of ROP datasets, label availability, and class distribution.

Task/Class	FARFUM-ROP	Ostrava	A-fundus	Overall
	*(n = 1,533)*	*(n = 6,004)*	*(n = 1,079)*	
Plus detection				
Normal	782 (51.0%)	5,375 (89.5%)	–	6,157 (81.7%)
Pre-plus	479 (31.2%)	–	–	479 (6.4%)
Plus	272 (17.7%)	629 (10.5%)	–	901 (12.0%)
Stage classification				
Stage 0	952 (62.1%)	4,086 (70.8%)	236 (21.9%)	5,274 (62.9%)
Stage 1	373 (24.3%)	252 (4.4%)	94 (8.7%)	719 (8.6%)
Stage 2	132 (8.6%)	458 (7.9%)	147 (13.6%)	737 (8.8%)
Stage 3	76 (5.0%)	125 (2.2%)	261 (24.2%)	462 (5.5%)
Stage 4	–	470 (8.1%)	–	470 (5.6%)
Laser scan	–	379 (6.6%)	341 (31.6%)	720 (8.6%)
Treatment decision				
No treatment	190 (15.4%)	–	–	190 (15.4%)
Revisit	593 (48.0%)	–	–	593 (48.0%)
Treatment	453 (36.7%)	–	–	453 (36.7%)

“–” indicates labels not available for that dataset. Percentages within each dataset column are computed with respect to the total number of images with valid labels for the corresponding task in that dataset. For FARFUM-ROP, stage and treatment labels are derived by expert voting with unresolved ties excluded; its expert stage fields contain Stage 1–3 only.

Due to the heterogeneous nature of the datasets, we implement several label alignment strategies to enable multi-task learning. For the Plus detection task, FARFUM-ROP provides three-class labels, while Ostrava only provides binary labels. We handle this by treating Ostrava samples with Plus disease as the Plus class and excluding them from Pre-Plus training. For the treatment decision task, only FARFUM-ROP provides treatment annotations; therefore, this task is supervised exclusively by FARFUM-ROP samples, while Ostrava and A-Fundus contribute only through shared feature learning and domain adaptation. Consequently, the treatment branch should be interpreted as an image-based auxiliary decision-support component rather than a fully externally validated treatment recommendation system. To prevent data leakage, we performed subject-level cross-validation for all three datasets using Group K-Fold (Liu and Carbonell, 2012). For FARFUM-ROP and Ostrava, patient identifiers are directly available in the dataset annotations. For A-Fundus, infant identifiers are provided in the accompanying metadata file released with the dataset, which maps each image to its corresponding infant ID and eye laterality. Using these identifiers, all images from the same subject were assigned exclusively to one subset among training, validation, and testing, ensuring that no two images from the same infant appear in different subsets. The Group K-Fold split guarantees subject-disjoint folds across all centers simultaneously.

### 3.2 Method overview

**FIGURE 1:**
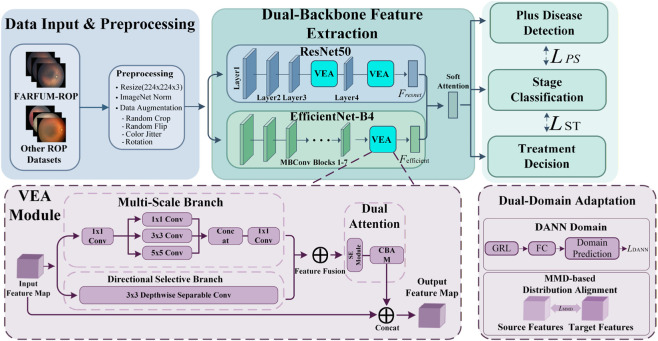
Overview of the VEA-Net architecture for multi-task ROP analysis. A retinal fundus image is processed by two complementary backbones: ResNet50, which emphasizes local vascular details, and EfficientNet-B4, which captures broader contextual representations. VEA modules are embedded into the feature extraction pathway to enhance vessel-related morphology through multi-scale convolution, directional selective filtering, and channel-spatial attention. The backbone features are projected to a shared dimension and fused by a soft-attention mechanism. The fused representation is then used by three task-specific heads for Plus disease detection, stage classification, and auxiliary treatment decision support. A domain adaptation module further regularizes the shared feature space using domain-adversarial learning and distribution alignment to reduce dataset-related feature bias.

VEA-Net is a dual-backbone multi-task learning framework designed for comprehensive ROP management. Figure 1 illustrates the overall architecture of VEA-Net, which consists of four main components: (1) a dual-backbone feature extraction module with embedded VEA modules, (2) a soft attention fusion mechanism, (3) three task-specific heads for Plus detection, stage classification, and treatment decision-making, and (4) a domain adaptation module for cross-center generalization. The network takes retinal fundus images as input and simultaneously outputs predictions for all three clinical tasks while learning domain-invariant representations. The dual-backbone design leverages the complementary strengths of ResNet50, which excels at capturing local vascular details through its residual learning framework, and EfficientNet-B4, which provides efficient multi-scale feature extraction through compound scaling. VEA modules are strategically inserted after selected mid-to-high-level feature stages of ResNet50 and after the final feature stage of EfficientNet-B4, enabling vascular enhancement at semantic feature levels while keeping the module placement consistent with the actual implementation.

The selection of ResNet50 and EfficientNet-B4 as the dual-backbone architecture was motivated by recent evidence in ROP-specific research. Mohiy et al. demonstrated that a hybrid ResNet50–EfficientNet-B4 architecture, termed ROPDeepX, achieves state-of-the-art performance on publicly available ROP benchmarks (Mohiy et al., 2026). Their study systematically evaluated individual backbones (VGG19, ResNet50, and EfficientNet-B4) as well as the hybrid combination, and found that integrating the two architectures yielded superior results across both multiclass and binary ROP classification tasks. Building on this finding, we adopted the same backbone pair for our multi-task framework. The complementary inductive biases of the two backbones are particularly well-suited to the visual characteristics of ROP. That is, ResNet50 captures fine-grained local vascular details through its residual learning framework, which is essential for detecting subtle vessel dilation and tortuosity that define Plus disease. EfficientNet-B4, with its compound scaling and squeeze-and-excitation mechanisms, provides broader multi-scale contextual representations that support stage classification and treatment decision-making.

### 3.3 Dual-backbone feature extraction

VEA-Net employs a dual-backbone architecture that combines ResNet50 and EfficientNet-B4 to leverage their complementary strengths in feature extraction. ResNet50, with its residual learning framework, excels at capturing fine-grained local vascular details through deep hierarchical representations. EfficientNet-B4, utilizing compound scaling and inverted residual blocks with squeeze-and-excitation connections, provides efficient multi-scale feature extraction with superior global context modeling. The ResNet50 backbone is initialized with ImageNet pre-trained weights and modified to remove the final fully-connected layer. The network produces four feature stages, named layer1 through layer4, with increasing receptive fields. VEA modules are inserted after layer3 and layer4 to enhance vascular features at both mid-level and high-level abstractions. The forward pass is given by Equation 1:

$$F_{\mathrm{resnet}}=\text{GlobalAvgPool}\left({\text{VEA}}_{N_{2}}\left(\text{layer}4\left({\text{VEA}}_{N_{1}}\left(\text{layer}3\left(\text{layer}2\left(\text{layer}1\left(X\right)\right)\right)\right)\right)\right)\right),$$ (1)

where $F_{\mathrm{resnet}}\in R^{N_{2}}$ represents the final $N_{2}$ -dimensional feature vector extracted by ResNet50, GlobalAvgPool denotes global average pooling that reduces spatial dimensions to a single vector, layer1 through layer4 represent the four residual stages of ResNet50 with increasing receptive fields, ${\text{VEA}}_{N_{1}}$ and ${\text{VEA}}_{N_{2}}$ denote VEA modules with $N_{1}$ and $N_{2}$ channels respectively that are inserted after layer3 and layer4, and X is the input retinal fundus image. Similarly, EfficientNet-B4 is initialized with ImageNet pre-trained weights and the classification head is removed. The backbone features module consists of seven sequential Mobile Inverted Bottleneck Convolution (MBConv) stages, denoted ${\text{MBConv}}_{1}$ through ${\text{MBConv}}_{7}$ , each with increasing expansion ratios and stride patterns. As in the ResNet50 pathway, a VEA module is inserted after the final feature stage to enhance vascular representations before pooling. The forward pass is given by Equation 2:

$$F_{\mathrm{efficient}}=\text{GlobalAvgPool}\left({\text{VEA}}_{N_{3}}\left({\text{MBConv}}_{7}\left({\text{MBConv}}_{6}\left(\ldots {\text{MBConv}}_{1}\left(X\right)\ldots \;\right)\right)\right)\right),$$ (2)

where $F_{\mathrm{efficient}}\in R^{N_{3}}$ represents the final $N_{3}$ -dimensional feature vector extracted by EfficientNet-B4, GlobalAvgPool denotes global average pooling, ${\text{VEA}}_{N_{3}}$ is the VEA module with $N_{3}$ channels applied after the final MBConv stage, ${\text{MBConv}}_{1}$ through ${\text{MBConv}}_{7}$ represent the seven Mobile Inverted Bottleneck Convolution stages that are composed sequentially, the ellipsis notation simply indicates the intermediate stages ${\text{MBConv}}_{2}$ through ${\text{MBConv}}_{6}$ (i.e., the output of each stage serves as the input to the next), and X is the input retinal fundus image.

### 3.4 Vascular enhancement attention (VEA) module

The hallmark of Plus disease, including vascular dilation, tortuosity, and abnormal posterior pole vessel morphology, manifests as subtle but clinically decisive changes in retinal vasculature. These abnormalities are not merely generic texture variations, they are characterized by elongated, directionally continuous, and locally curved vessel structures. Standard convolutional operations may capture these patterns implicitly, but they do not explicitly emphasize the directional continuity and curvature variations that are central to Plus disease assessment. Therefore, we designed the VEA module to enhance vascular morphology through three complementary mechanisms: multi-scale feature extraction, directional selective filtering, and dual attention. The multi-scale branch captures vessels with different calibers and local contextual ranges, the directional selective branch strengthens orientation-sensitive vascular responses, and the attention mechanism suppresses irrelevant background regions while highlighting clinically meaningful vascular structures.

#### 3.4.1 Multi-scale convolution branch

Retinal vessels vary substantially in caliber, ranging from thin peripheral branches to relatively thick posterior pole vessels. In ROP, pathological dilation and tortuosity may appear at different spatial scales. We therefore employ three parallel convolution paths to capture vascular features at multiple receptive fields. Given an input feature map $X\in R^{C\times H\times W}$ , where C denotes the number of input channels and H and W denote the spatial dimensions, the multi-scale branch first projects features to an intermediate dimension $C^{\prime }=\max\left(C/4,32\right)$ to reduce computational cost. The three parallel paths consist of a 1×1 convolution for local channel mixing, a 3×3 depthwise convolution for short-range vessel orientation and curvature patterns, and a 5×5 depthwise convolution with padding = 2 for larger vascular context. Each path is followed by batch normalization and ReLU activation. The outputs are concatenated along the channel dimension and fused through a 1×1 convolution. The multi-scale feature extraction formula is as follows:

$$F_{ms}={\text{Conv}}_{1\times 1}\left(\text{BN}\left(\text{ReLU}\left(\text{Concat}\left(P_{1}\left(X\right),P_{3}\left(X\right),P_{5}\left(X\right)\right)\right)\right)\right),$$ (3)

where $F_{ms}$ represents the multi-scale fused feature map, ${\text{Conv}}_{1\times 1}$ denotes the 1×1 convolution operation for channel fusion, BN represents batch normalization, ReLU is the rectified linear unit activation function, Concat indicates concatenation along the channel dimension, and $P_{k}$ denotes the k×k convolution path for k∈{1,3,5} .

#### 3.4.2 Directional selective convolution

To enhance the tortuous and dilated vessel patterns that characterize Plus disease, we introduce a direction-sensitive depthwise convolution branch. Retinal vessels are thin, elongated structures with strong local orientation continuity. Vessel tortuosity corresponds to rapid local orientation changes along the vessel trajectory, whereas vascular dilation is reflected by increased vessel caliber along such oriented structures. Depthwise convolution operates independently on each channel and therefore preserves channel-specific orientation responses instead of mixing them prematurely. This design allows the network to emphasize vessel-like directional patterns and local curvature changes while maintaining a low parameter cost. The directional selective convolution formula is as follows:

$$F_{\mathrm{dir}}=\text{BN}\left(\text{ReLU}\left({\text{DWConv}}_{3\times 3}\left(X\right)\right)\right),$$ (4)

where $F_{\mathrm{dir}}$ denotes the directionally enhanced feature map, ${\text{DWConv}}_{3\times 3}$ represents the 3×3 depthwise convolution operation applied independently to each channel, BN is batch normalization, and ReLU is the activation function. By preserving channel-wise directional responses, this branch is particularly suitable for highlighting abnormal vascular trajectories and caliber changes in ROP fundus images.

The design of directional selective convolution is motivated by the vascular morphology of ROP. Plus disease is clinically characterized by abnormal posterior pole vascular dilation and tortuosity, while disrupted retinal vascular development and oxygen-related vascular regression are central to neonatal retinal vascular pathology (Chiang et al., 2021; Xu et al., 2025). Directional filtering is therefore suitable for enhancing elongated and curvilinear structures because retinal vessels appear as oriented tubular patterns rather than isolated texture elements. By aggregating responses along multiple directions, the VEA module is designed to improve sensitivity to vessel trajectory changes, local curvature, and caliber-related patterns that are relevant to Plus disease assessment.

#### 3.4.3 Feature fusion

The multi-scale and directional features are combined through element-wise addition. The feature fusion is given by Equation 5:

$$F_{\mathrm{enhanced}}=F_{ms}+F_{\mathrm{dir}},$$ (5)

where $F_{\mathrm{enhanced}}$ represents the enhanced feature map after fusion, $F_{ms}$ denotes the multi-scale features from Equation 3, $F_{\mathrm{dir}}$ represents the directionally selective features from Equation 4, and + indicates element-wise addition.

#### 3.4.4 Dual attention mechanism

We apply a two-stage attention mechanism to further refine vascular features. A Squeeze-and-Excitation (SE) block performs channel-wise recalibration. The SE attention is given by Equation 6:

$$F_{se}=F_{\mathrm{enhanced}}⊙\sigma \left({\text{FC}}_{2}\left(\text{ReLU}\left({\text{FC}}_{1}\left(\text{GAP}\left(F_{\mathrm{enhanced}}\right)\right)\right)\right)\right),$$ (6)

where $F_{se}$ denotes the feature map after SE attention, ⊙ represents element-wise multiplication, σ is the sigmoid activation function that produces channel-wise attention weights in the range [0,1] , GAP denotes global average pooling that compresses spatial information into channel descriptors, ${\text{FC}}_{1}$ and ${\text{FC}}_{2}$ are fully connected layers with reduction ratio r=16 that learn channel dependencies, where ${\text{FC}}_{1}$ reduces the channel dimension by factor r and ${\text{FC}}_{2}$ restores it to the original dimension. Then, a CBAM spatial attention module computes a spatial attention map. The spatial attention map is computed in Equation 7:

$$M_{s}=\sigma \left({\text{Conv}}_{7\times 7}\left(\left[\text{AvgPool}\left(F_{se}\right);\text{MaxPool}\left(F_{se}\right)\right]\right)\right).$$ (7)

The spatial attention application formula is as follows:

$$F_{\mathrm{cbam}}=F_{se}⊙M_{s},$$ (8)

where $M_{s}$ represents the spatial attention map, σ denotes the sigmoid activation function, ${\text{Conv}}_{7\times 7}$ is a 7×7 convolution operation that generates the attention weights, [⋅;⋅] indicates concatenation along the channel dimension, AvgPool and MaxPool represent average pooling and max pooling operations that capture complementary spatial information, $F_{\mathrm{cbam}}$ denotes the feature map after CBAM spatial attention, and ⊙ is element-wise multiplication that applies the spatial attention weights to the input features. The final output incorporates a residual connection to preserve gradient flow. The residual connection is given by Equation 9:

$$F_{\mathrm{out}}=F_{\mathrm{cbam}}+X,$$ (9)

where $F_{\mathrm{out}}$ represents the final output feature map of the VEA module, $F_{\mathrm{cbam}}$ denotes the feature map after CBAM spatial attention from Equation 8, X is the original input feature map, and + indicates element-wise addition that enables the residual connection for preserving gradient flow during backpropagation. VEA modules are strategically embedded within the dual-backbone architecture. In the ResNet50 pathway, VEA modules with N_1 channels are inserted after layer3, and modules with N_2 channels are inserted after layer4. In the EfficientNet-B4 pathway, a single VEA module with N_3 channels is applied after the final feature pyramid stage. This placement allows the network to progressively enhance vascular features at multiple levels of abstraction, from fine-grained local patterns to coarse-grained global context.

### 3.5 Hierarchical multi-task learning

The clinical workflow for ROP management inherently involves multiple interrelated decisions: detecting the presence of Plus disease, determining the disease stage, and recommending appropriate treatment. These tasks are not independent but rather form a hierarchical decision chain where earlier assessments inform later clinical judgments. Traditional approaches that treat these tasks separately fail to leverage these natural correlations, potentially leading to inconsistent predictions and suboptimal performance. Our hierarchical multi-task learning framework addresses this limitation by jointly optimizing all three tasks while explicitly modeling their clinical relationships through shared representations and consistency constraints. Specifically, we formulate ROP management as a three-task learning problem that simultaneously addresses Plus detection, stage classification, and treatment decision. These three tasks reflect the complete clinical workflow from initial screening through final treatment recommendation, enabling the model to provide comprehensive diagnostic support rather than isolated predictions.

#### 3.5.1 Soft attention feature fusion

Features from ResNet50 $\left(F_{\mathrm{resnet}}\in R^{2048}\right)$ and EfficientNet-B4 $\left(F_{\mathrm{efficient}}\in R^{1792}\right)$ are fused through a learnable soft attention mechanism. First, both feature vectors are projected to a common dimension D=2048 . The feature projection formula is as follows:

$${\tilde{F}}_{\mathrm{resnet}}={\text{Linear}}_{D_{1}}\left(F_{\mathrm{resnet}}\right),\;{\tilde{F}}_{\mathrm{efficient}}={\text{Linear}}_{D_{2}}\left(F_{\mathrm{efficient}}\right),$$ (10)

where ${\tilde{F}}_{\mathrm{resnet}}$ and ${\tilde{F}}_{\mathrm{efficient}}$ represent the projected feature vectors from ResNet50 and EfficientNet-B4 respectively, both with dimension D=2048 , ${\text{Linear}}_{D_{1}}$ and ${\text{Linear}}_{D_{2}}$ denote linear transformation layers that project the original features to the common dimension D , $F_{\mathrm{resnet}}$ is the 2048-dimensional feature vector from ResNet50, and $F_{\mathrm{efficient}}$ is the 1792-dimensional feature vector from EfficientNet-B4. The attention weights are computed by concatenating the projected features and passing them through a multi-layer perceptron. The attention weight computation formula is as follows:

$$\left[\alpha_{1},\alpha_{2}\right]=\text{Softmax}\left({\text{Linear}}_{2}\left(\text{ReLU}\left({\text{Linear}}_{D}\left(\text{Concat}\left({\tilde{F}}_{\mathrm{resnet}},{\tilde{F}}_{\mathrm{efficient}}\right)\right)\right)\right)\right),$$ (11)

where $\left[\alpha_{1},\alpha_{2}\right]$ represents the learned attention weights for ResNet50 and EfficientNet-B4 respectively, with $\alpha_{1}+\alpha_{2}=1$ , Softmax is the softmax function that normalizes the weights to sum to 1, ${\text{Linear}}_{2}$ is a linear layer that outputs 2 attention scores, ReLU is the rectified linear unit activation, ${\text{Linear}}_{D}$ is a linear layer that processes the concatenated features, Concat denotes concatenation of the two projected feature vectors, and ${\tilde{F}}_{\mathrm{resnet}}$ and ${\tilde{F}}_{\mathrm{efficient}}$ are the projected features from Equation 10. The fused representation is a weighted combination. The feature fusion formula is as follows:

$$F_{\mathrm{fused}}=\alpha_{1}\cdot {\tilde{F}}_{\mathrm{resnet}}+\alpha_{2}\cdot {\tilde{F}}_{\mathrm{efficient}},$$ (12)

where $F_{\mathrm{fused}}$ represents the final fused feature vector with dimension D=2048 , $\alpha_{1}$ and $\alpha_{2}$ are the attention weights from Equation 11, ${\tilde{F}}_{\mathrm{resnet}}$ and ${\tilde{F}}_{\mathrm{efficient}}$ are the projected features, and ⋅ denotes scalar multiplication. This soft attention mechanism allows the network to dynamically adjust the contribution of each backbone based on the input image characteristics, learning to rely more on ResNet50 for local vascular details and EfficientNet-B4 for global context when appropriate.

#### 3.5.2 Task-specific heads

Three independent task heads are attached to the fused features, each implementing a two-layer fully-connected network with dropout regularization. The task head computation is given by Equation 13:

$$H_{t}={\text{Linear}}_{C_{t}}\left(\text{Dropout}\left(\text{ReLU}\left({\text{Linear}}_{512}\left(F_{\mathrm{fused}}\right)\right)\right)\right),$$ (13)

where $H_{t}$ represents the output logits for task t , t∈{Plus, stage, treatment} denotes the three clinical tasks (Plus detection, stage classification, and treatment decision), $C_{t}$ is the number of output classes for task t with $C_{\text{Plus}}=3$ (i.e., Normal, Pre-Plus, Plus), $C_{\text{stage}}=6$ (i.e., Stage 0–5), and $C_{\text{treatment}}=3$ (i.e., No Treatment, Revisit, Treatment), ${\text{Linear}}_{512}$ is a fully-connected layer that projects the fused features to 512 dimensions, ReLU is the activation function, Dropout is the dropout regularization layer that prevents overfitting, ${\text{Linear}}_{C_{t}}$ is the output layer that produces $C_{t}$ logits, and $F_{\mathrm{fused}}$ is the fused feature vector from Equation 12. Each head outputs logits that are converted to class probabilities via softmax.

#### 3.5.3 Task correlation loss

To model clinical relationships between tasks, we introduce a task correlation loss. The purpose of this loss is not to impose deterministic diagnostic rules, but to penalize clinically implausible combinations among Plus status, stage prediction, and treatment recommendation.

Plus-Stage Consistency: When Plus disease is present, higher stages (Stage 3 and above) are more likely. We implement this relationship as a soft probabilistic constraint between the predicted Plus and stage distributions. Specifically, let $p_{\text{Plus}}$ denote the predicted probability of Plus disease, and $p_{\text{high}}$ denote the predicted probability mass on high stages. The Plus-Stage consistency loss encourages high $p_{\text{Plus}}$ to co-occur with high $p_{\text{high}}$ :

$$L_{PS}=-\gamma_{PS}\cdot E\left[\;p_{\text{Plus}}\cdot \log\left(p_{\text{high}}+\epsilon \right)\;\right],$$ (14)

where $\gamma_{PS}=0.5$ scales the contribution of this term, and ϵ is a small constant for numerical stability. This loss does not enforce a hard rule, it provides a soft signal that reinforces the natural association between Plus disease and advanced stages.

Stage-Treatment Consistency: Treatment decisions should be consistent with stage severity. We define three mutually complementary soft constraints based on the predicted stage and treatment probability distributions. Let $p_{\text{low}}$ , $p_{\text{mid}}$ , and $p_{\text{high}}$ denote the predicted probability masses on Stage 0–1, Stage 2, and Stage 3–5, respectively, and let $q_{\text{no}}$ , $q_{\text{revisit}}$ , and $q_{\text{yes}}$ denote the predicted probabilities of No Treatment, Revisit, and Treatment. The Stage-Treatment consistency loss is:

$$L_{ST}=\gamma_{ST}\cdot (E\left[-p_{\text{low}}\log\left(q_{\text{no}}+\epsilon \right)\right]+E\left[-p_{\text{mid}}\log\left(q_{\text{revisit}}+\epsilon \right)\right]+E\left[-p_{\text{high}}\log\left(q_{\text{yes}}+\epsilon \right)\right]),$$ (15)

where $\gamma_{ST}=0.5$ . These three terms jointly encourage low stages to align with No Treatment, intermediate stages with Revisit, and high stages with active Treatment, without overriding the supervised treatment labels. The total task correlation loss is:

$$L_{\text{correlation}}=\lambda_{PS}L_{PS}+\lambda_{ST}L_{ST},$$ (16)

where $L_{\text{correlation}}$ represents the total task correlation loss, $\lambda_{PS}$ and $\lambda_{ST}$ are hyperparameters that control the relative importance of the Plus-Stage and Stage-Treatment consistency losses, respectively, $L_{PS}$ is the Plus-Stage consistency loss from Equation 14, and $L_{ST}$ is the Stage-Treatment consistency loss from Equation 15.

#### 3.5.4 Task auxiliary loss

Beyond the pairwise consistency constraints above, we introduce an auxiliary loss that leverages the predicted Plus status to reinforce the stage prediction. Let $p_{\text{abnormal}}^{\text{Plus}}$ be the predicted probability of abnormal Plus status (Pre-Plus or Plus), and $p_{\text{abnormal}}^{\text{stage}}$ be the predicted probability of abnormal stage (Stage 1–5). The auxiliary loss is defined as:

$$L_{\text{aux}}=\gamma_{\text{aux}}\cdot E\left[\;-p_{\text{abnormal}}^{\text{Plus}}\cdot \log\left(p_{\text{abnormal}}^{\text{stage}}+\epsilon \right)\;\right],$$ (17)

where $\gamma_{\text{aux}}=0.3$ . This term provides an additional inductive bias: when the network predicts abnormal Plus disease, it should simultaneously assign higher probability to non-normal stages.

### 3.6 Dual-domain adaptation

A critical challenge in deploying ROP diagnostic models across diverse clinical settings is the domain shift caused by variations in imaging equipment, acquisition protocols, image quality, and patient populations. Models trained on data from one center may therefore learn center-specific appearance patterns rather than disease-relevant vascular morphology. To address this issue, we combine domain-adversarial learning with explicit distribution alignment because the two mechanisms regularize different aspects of domain shift. The domain-adversarial component encourages the shared representation to be non-discriminative with respect to dataset origin, thereby reducing global center-specific information through a discriminative objective. In contrast, the MMD component directly minimizes the statistical discrepancy between source and target feature distributions, providing an explicit moment-level alignment constraint. These two mechanisms are complementary: DANN promotes global domain confusion, whereas MMD stabilizes feature distribution alignment at the batch level. This dual-domain adaptation strategy is designed to learn disease-relevant and domain-invariant representations across FARFUM-ROP, Ostrava, and A-Fundus without requiring treatment labels from all centers.

### 3.6.1 Domain-adversarial neural networks (DANN)

To learn domain-invariant features across the three datasets, we employ a domain classifier with gradient reversal. The domain classifier D is a three-layer fully-connected network. The domain classifier is defined in Equation 18:

$$D\left(F_{\mathrm{fused}}\right)={\text{Linear}}_{3}\left(\text{Dropout}\left(\text{ReLU}\left({\text{Linear}}_{128}\left(\text{Dropout}\left(\text{ReLU}\left({\text{Linear}}_{256}\left(F_{\mathrm{fused}}\right)\right)\right)\right)\right)\right)\right),$$ (18)

where $D\left(F_{\mathrm{fused}}\right)$ represents the output domain prediction with 3 classes (FARFUM, Ostrava, A-Fundus), ${\text{Linear}}_{256}$ , ${\text{Linear}}_{128}$ , and ${\text{Linear}}_{3}$ are fully-connected layers that project the input to 256, 128, and 3 dimensions respectively, ReLU is the activation function applied after each linear layer, Dropout is the dropout regularization layer with dropout rate 0.5, and $F_{\mathrm{fused}}$ is the fused feature vector from Equation 12. During training, the gradient reversal layer (GRL) negates gradients flowing from the domain classifier to the feature extractor. The gradient reversal is given by Equation 19:

$$\frac{\partial L_{\text{DANN}}}{\partial F_{\mathrm{fused}}}=-\lambda_{\text{da}}\cdot \frac{\partial L_{\text{domain}}}{\partial F_{\mathrm{fused}}},$$ (19)

where $\frac{\partial L_{\text{DANN}}}{\partial F_{\mathrm{fused}}}$ represents the gradient of the DANN loss with respect to the fused features, $\lambda_{\text{da}}$ is a hyperparameter that controls the strength of domain adaptation, $L_{\text{domain}}$ is the domain classification loss, $F_{\mathrm{fused}}$ is the fused feature vector, and the negative sign indicates that gradients are reversed during backpropagation to encourage domain-invariant feature learning. The domain classification loss is:

$$L_{\text{DANN}}=-\frac{1}{N}\overset{N}{\underset{i=1}{\sum }}\log D\left(F_{\mathrm{fused}}^{\left(i\right)}\right)\left[d_{i}\right],$$ (20)

where $L_{\text{DANN}}$ denotes the domain classification loss, N is the batch size, $F_{\mathrm{fused}}^{\left(i\right)}$ represents the fused feature vector for the i -th sample in the batch, $D\left(F_{\mathrm{fused}}^{\left(i\right)}\right)$ is the predicted domain probability distribution for sample i , $d_{i}\in \left\{0,1,2\right\}$ indicates the ground truth domain label for sample i (0 for FARFUM, 1 for Ostrava, 2 for A-Fundus), and [⋅] denotes indexing to select the probability of the correct domain class.

### 3.6.2 Maximum mean discrepancy (MMD)

In addition to adversarial training, we explicitly minimize the distribution discrepancy between source and target domains using Maximum Mean Discrepancy (MMD). The MMD measures the distance between feature distributions in a reproducing kernel Hilbert space (RKHS). The MMD is defined in Equation 21:

$${\text{MMD}}^{2}={\left\|\frac{1}{n_{s}}\overset{n_{s}}{\underset{i=1}{\sum }}\phi \left(F_{s}^{\left(i\right)}\right)-\frac{1}{n_{t}}\overset{n_{t}}{\underset{j=1}{\sum }}\phi \left(F_{t}^{\left(j\right)}\right)\right\|}_{H}^{2},$$ (21)

where ${\text{MMD}}^{2}$ denotes the squared Maximum Mean Discrepancy, $n_{s}$ and $n_{t}$ represent the number of samples in the source and target domains respectively, $F_{s}^{\left(i\right)}$ is the i -th feature sample from the source domain, $F_{t}^{\left(j\right)}$ is the j -th feature sample from the target domain, ϕ(⋅) is the feature mapping function that maps samples to the RKHS H , and $\|\cdot {\|}_{H}$ denotes the norm in the RKHS. In practice, we employ a multi-scale Gaussian RBF kernel to capture distribution discrepancies at multiple similarity levels. The Gaussian RBF kernel is defined as $k\left(x_{i},x_{j}\right)=\exp\left(-\|x_{i}-x_{j}{\|}^{2}/h\right)$ , where h controls the kernel bandwidth. We use K=5 kernels with bandwidths $\left\{h\cdot 2^{i}|i=0,\ldots ,K-1\right\}$ and average their outputs to improve stability across different feature scales. The resulting MMD loss is computed as:

$$L_{\text{MMD}}=\frac{1}{n_{s}^{2}}\overset{n_{s}}{\underset{i,j}{\sum }}k\left(F_{s}^{\left(i\right)},F_{s}^{\left(j\right)}\right)+\frac{1}{n_{t}^{2}}\overset{n_{t}}{\underset{i,j}{\sum }}k\left(F_{t}^{\left(i\right)},F_{t}^{\left(j\right)}\right)-\frac{2}{n_{s}n_{t}}\overset{n_{s},n_{t}}{\underset{i,j}{\sum }}k\left(F_{s}^{\left(i\right)},F_{t}^{\left(j\right)}\right),$$ (22)

where $L_{\text{MMD}}$ represents the MMD loss, k(⋅,⋅) denotes the multi-scale Gaussian RBF kernel (averaged over K=5 bandwidths with base bandwidth h=2.0 ), the first term computes the average kernel similarity between source domain samples, the second term computes the average similarity between target domain samples, and the third term computes the negative average similarity between source and target domain samples. The MMD is computed on the features between source domain and target domain.

### 3.7 Overall loss function

The design of our overall loss function is motivated by the need to balance multiple objectives: accurate multi-task prediction, clinical consistency across related tasks, and robust domain generalization. Each component serves a specific purpose while working synergistically with others. The task-specific losses ensure individual task performance, the hierarchical consistency losses enforce clinical relationships between tasks, and the domain adaptation losses promote feature invariance across centers. Dynamic weighting further adapts to the varying convergence rates of different tasks during training. The total training objective combines all components. The overall loss function is defined in Equation 23:

$$L_{\text{total}}=\lambda_{1}L_{\text{plus}}+\lambda_{2}L_{\text{stage}}+\lambda_{3}L_{\text{treatment}}+\lambda_{4}L_{\text{correlation}}+\lambda_{5}L_{\text{aux}}+\lambda_{6}L_{\text{DANN}}+\lambda_{7}L_{\text{MMD}},$$ (23)

where $L_{\text{total}}$ represents the total training loss, and $\lambda_{1}$ through $\lambda_{7}$ are the weighting hyperparameters for each loss component. Here, $L_{\text{plus}}$ and $L_{\text{treatment}}$ are focal losses with γ=2 to handle class imbalance for Plus detection and treatment decision tasks, $L_{\text{stage}}$ is a weighted cross-entropy loss with class-specific weights [1.0,1.5,1.2,2.0,3.0,2.5] to address the imbalance across the six stage classes, $L_{\text{correlation}}$ is the task correlation loss from Equation 16 that decomposes into Plus-Stage and Stage-Treatment consistency terms, $L_{\text{aux}}$ is the task auxiliary loss from Equation 17 that leverages the predicted abnormal Plus probability to reinforce the predicted abnormal stage probability, $L_{\text{DANN}}$ is the domain adversarial loss defined in Equation 20, and $L_{\text{MMD}}$ is the Maximum Mean Discrepancy loss defined in Equation 22 using a multi-scale Gaussian RBF kernel.

### 3.8 Training strategy

Our training strategy employs 10-fold Group K-Fold cross-validation to ensure robust performance estimation while preventing patient-level data leakage. In Group K-Fold, the splitting unit is the subject rather than the individual image. That is, all images belonging to the same subject are kept together in a single fold. Across our three datasets, this corresponds to 737 unique subjects. The overall procedure is outlined in Algorithm 1, which integrates multi-task learning, domain adaptation, and early stopping mechanisms. Key implementation details include: (1) cosine annealing learning rate schedule that gradually reduces the learning rate from $10^{-4}$ ; (2) comprehensive data augmentation including random resized crop to 224×224 , random horizontal and vertical flips, and color jittering with brightness and contrast variations of 0.2; and (3) batch size of 16 with AdamW optimizer (weight decay = $10^{-4}$ ). The best model weights from each fold are saved and used for final test evaluation.

Algorithm 1 **VEA-Net Training Procedure with 10-Fold Group K-Fold Cross-Validation.**

**Require:** Dataset $D=D_{\text{FARFUM}}\cup D_{\text{Ostrava}}\cup D_{\text{A}-\text{Fundus}}$
  
**Require:** Number of folds K=10 , epochs E=30 , batch size B=16

**Require:** Learning rate $\eta =10^{-4}$ , early stopping patience P=5

**Require:** Subject identifiers G for all images (patient IDs for FARFUM/Ostrava, infant IDs for A-Fundus)
**Ensure:** Best model weights $\theta^{*}$

1: Split D into K subject-disjoint folds $\left\{F_{1},\ldots ,F_{K}\right\}$ using Group K-Fold on G

2: **for** fold k=1 to K **do**
3: $D_{\text{test}}^{\left(k\right)}\leftarrow F_{k}$

4: $D_{\text{remain}}^{\left(k\right)}\leftarrow {⋃}_{j\neq k}F_{j}$

5:  Split $D_{\text{remain}}^{\left(k\right)}$ into $D_{\text{train}}^{\left(k\right)}$ and $D_{\text{val}}^{\left(k\right)}$ for training and early stopping
6:  Initialize VEA-Net M with ImageNet weights
7: $\theta^{*}\leftarrow \text{None}$ , best_acc ←0 , patience_counter ←0

8:  **for** epoch e=1 to E **do**
9:   **for** each batch $B\subset D_{\text{train}}^{\left(k\right)}$ **do**
10:    Compute forward pass: O←M(B)

11:    Compute loss: $L\leftarrow L_{\text{total}}\left(O,B\right)$

12:    Backpropagate and update: $\theta \leftarrow \theta -\eta \nabla_{\theta }L$

13:   **end for**
14:   Evaluate on $D_{\text{val}}^{\left(k\right)}$ : $\text{acc}\leftarrow \frac{1}{3}\underset{t}{\sum }{\text{Acc}}_{t}$

15:   **if** acc > best_acc +0.001 **then**
16: $\theta^{*}\leftarrow \theta$ , best_acc ← acc, patience_counter ←0

17:   **else**
18:    patience_counter ← patience_counter +1

19:    **end if**
20:   **if** patience_counter ≥P **then**
21:    **break** (early stopping)
22:   **end if**
23:  **end for**
24: $M^{*}\leftarrow M\left(\theta^{*}\right)$

25:  Evaluate on $D_{\text{test}}^{\left(k\right)}$ : ${\text{metrics}}_{k}\leftarrow \left\{{\text{Acc}}_{\text{Plus}},{\text{Acc}}_{\text{stage}},{\text{Acc}}_{\text{treatment}}\right\}$

26: **end for**
27: Return average metrics: $\bar{\text{metrics}}\leftarrow \frac{1}{K}\overset{K}{\underset{k=1}{\sum }}{\text{metrics}}_{k}$

### 3.9 Evaluation metrics

We evaluate model performance using multiple metrics to provide a comprehensive assessment of clinical utility. The primary metric is accuracy, computed as the proportion of correctly classified samples among those with valid labels. For each task t∈{Plus, stage, treatment} , accuracy is calculated. The accuracy is computed as in Equation 24:

$$\text{Accuracy}=\frac{1}{N_{t}}\overset{N_{t}}{\underset{i=1}{\sum }}I\left({\hat{y}}_{i}^{\left(t\right)}=y_{i}^{\left(t\right)}\right),$$ (24)

where Accuracy represents the accuracy for task t , $N_{t}$ is the number of samples with valid labels for task t , I(⋅) is the indicator function that equals 1 when the predicted label matches the ground truth and 0 otherwise, ${\hat{y}}_{i}^{\left(t\right)}$ is the predicted class for the i -th sample on task t , $y_{i}^{\left(t\right)}$ is the ground truth label for the i -th sample on task t , and the summation counts the number of correctly classified samples. Samples with label = −1 (indicating missing annotations) are excluded from the calculation.

In addition to accuracy, we report the Area Under the Receiver Operating Characteristic Curve (AUROC) to provide a threshold-independent measure of discriminative ability. For binary classification tasks, AUROC is computed directly from the predicted probability of the positive class. For multi-class tasks, we adopt the one-vs-rest (OvR) strategy: for each class c∈{1,…,C} , a binary AUROC is computed by treating class c as positive and all other classes as negative, and the final multi-class AUROC is the unweighted average across all classes, as defined in Equation 25:

$$\text{AUROC}=\frac{1}{C}\overset{C}{\underset{c=1}{\sum }}{\text{AUROC}}_{c},$$ (25)

where ${\text{AUROC}}_{c}$ is the binary AUROC for class c under the one-vs-rest formulation, computed using the trapezoidal rule over the ROC curve constructed from the predicted probabilities and ground truth labels.

## 4 Experimental results

### 4.1 Model implementation

VEA-Net was implemented using PyTorch 1.12 and trained on 2 × NVIDIA GeForce RTX 4090 D GPUs (48 GB VRAM each). The ResNet50 backbone was initialized with ImageNet pre-trained weights using torchvision’s official implementation, while EfficientNet-B4 was also initialized with ImageNet pre-trained weights from the same source. All other layers were initialized using Xavier initialization. Training was conducted with a batch size of 16, initial learning rate of $10^{-4}$ , and AdamW optimizer with weight decay of $10^{-4}$ . A cosine annealing schedule was employed to gradually reduce the learning rate during training. The maximum number of epochs was set to 30, with early stopping triggered when validation performance failed to improve for 5 consecutive epochs. Images were preprocessed by resizing to 224×224 pixels and normalized using ImageNet statistics. Data augmentation during training included random resized crop, random horizontal and vertical flips, and color jittering. No augmentation was applied during validation and testing.

### 4.2 Overall performance on three clinical tasks

We conducted 10-fold cross-validation to evaluate VEA-Net’s performance across all three clinical tasks. Table 2 summarizes the validation and independent test results. VEA-Net achieved test accuracy of 85.44% ± 15.78% for Plus detection, 74.81% ± 10.17% for stage classification, and 56.80% ± 23.48% for treatment decision-making, with corresponding test AUROC values of 91.76% ± 4.16%, 88.26% ± 4.61%, and 66.81% ± 15.87%. The highest and most stable performance was observed for Plus detection, which is consistent with the fact that Plus disease is directly associated with visually prominent posterior pole vascular dilation and tortuosity (Chiang et al., 2021). Stage classification was more challenging because it requires recognition of broader retinal anatomical changes and disease progression patterns.

**TABLE 2:** Overall performance on validation and test sets.

Task	Val. Acc.	Test Acc.	Test AUROC
Plus detection	86.18 ± 8.66	85.44 ± 15.78	91.76 ± 4.16
Stage classification	76.32 ± 6.93	74.81 ± 10.17	88.26 ± 4.61
Treatment decision	56.14 ± 16.11	56.80 ± 23.48	66.81 ± 15.87

Values are mean ± standard deviation.

For Plus detection, the standard deviation of 15.78% is primarily driven by the heterogeneous class distributions across folds. Ostrava contributes 89.5% Normal samples, and the proportion of Ostrava versus FARFUM-ROP samples in each fold’s test set varies, causing accuracy fluctuations when the test set shifts toward the more imbalanced Ostrava distribution. For stage classification, the standard deviation of 10.17% reflects the inherent difficulty of distinguishing six clinically defined stages, compounded by the uneven distribution of advanced-stage samples across folds. For treatment decision-making, the largest standard deviation of 23.48% has three contributing factors: (1) treatment labels are available only in FARFUM-ROP, which contains just 68 unique patients; under 10-fold subject-level splitting, each test fold includes approximately 7 patients with treatment labels, meaning a single misclassification can shift the fold’s treatment accuracy by more than 14%; (2) the three treatment classes are moderately imbalanced, and the boundary between Revisit and Treatment is clinically ambiguous; (3) treatment recommendations in real clinical practice depend on variables beyond fundus appearance, including gestational age, birth weight, postmenstrual age, and local protocols, which are not available in the current dataset.

**FIGURE 2:**
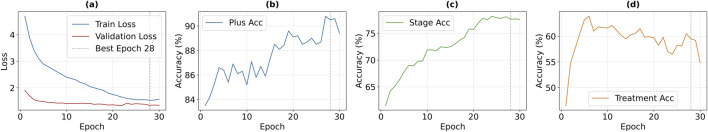
Training convergence curves of VEA-Net and validation accuracy changes across the clinical tasks. **(a)** Training loss and validation loss over epochs, where training loss continues to decrease and validation loss stabilizes after an early decline; **(b)** Validation accuracy for the Plus detection task shows an overall upward trend and reaches a high level in the later training stages; **(c)** Validation accuracy for the stage classification task gradually improves and enters a relatively stable plateau in the later stages; **(d)** Validation accuracy for the treatment decision task increases rapidly in early epochs and then exhibits considerable fluctuations. The gray dashed line indicates the mean best epoch across the 10-fold cross-validation, approximately epoch 28.

Figure 2 illustrates the training convergence behavior of VEA-Net and the validation accuracy changes across the three clinical tasks. Overall, the training loss decreases rapidly within the first several epochs and then maintains a sustained, smooth downward trend, indicating that the model effectively optimizes its parameters. The validation loss drops significantly in the early training phase and subsequently enters a low-level plateau with mild fluctuations, suggesting that the model gradually converges in later epochs without showing significant sustained overfitting. For the individual tasks, the validation accuracy for Plus detection shows an overall upward trend and reaches its highest level in the later training stages, indicating that the model can stably learn Plus-related vascular morphological features. The accuracy of the stage classification task gradually improves and stabilizes after approximately epoch 20, reflecting the model’s progressively strengthening ability to discriminate disease stage features. In contrast, the treatment decision task shows a rapid increase in accuracy during the early training phase but is followed by notable fluctuations and a slight decline in the final epochs, suggesting that this task is more strongly affected by clinical decision complexity, class boundaries, and multi-task optimization trade-offs. Across all tasks, the mean best epoch across the 10 folds is located around epoch 28, confirming that a validation-based model selection strategy helps achieve better generalization performance.

### 4.3 Ablation study

To quantify the contribution of each component in VEA-Net, we conducted an ablation study by removing individual modules and measuring the performance impact under a 10-fold Group K-Fold patient-level split across all three datasets. Table 3 summarizes the per-task ACC and AUROC results. As shown in Table 3, the full VEA-Net configuration provides a favorable overall balance across the evaluated metrics. Removing the VEA module causes the most notable performance drop in Treatment AUROC, while Plus and Stage tasks remain relatively stable. This per-task analysis indicates that the vascular enhancement attention module is particularly beneficial for treatment-relevant feature discrimination. Removing the dual-backbone design (retaining only ResNet50) reduces Plus AUROC by 3.61% (from 91.76% to 88.15%) and Treatment AUROC by 3.97% (from 66.81% to 62.84%), confirming the value of combining ResNet50’s local feature extraction with EfficientNet-B4’s global context modeling. Removing task correlation losses reduces Plus AUROC by 2.68% (from 91.76% to 89.08%), Stage AUROC by 0.32% (from 88.26% to 87.94%), and Treatment AUROC by 4.36% (from 66.81% to 62.45%), while Plus and Stage tasks remain largely unaffected, validating that hierarchical consistency regularization is most impactful for the treatment decision task. Removing domain adaptation entirely (w/o Domain Adapt.) yields a similar Plus AUROC (91.71% vs. 91.76%) and a higher Treatment AUROC, but it lacks the cross-center feature-regularization mechanism provided by DANN + MMD and should therefore be interpreted cautiously. Regarding the two domain adaptation components individually, the MMD-only configuration yields Plus AUROC of 88.27%, Stage AUROC of 88.14%, and Treatment AUROC of 60.36%; the DANN-only configuration yields Plus AUROC of 86.47%, Stage AUROC of 81.76%, and Treatment AUROC of 60.27%. Both strategies are effective individually, but their combination yields the best per-task AUROC across all three tasks, confirming that DANN (adversarial domain confusion) and MMD (distribution-level alignment) regularize complementary aspects of domain shift. Finally, the Baseline (ResNet50 without VEA, dual-backbone, or domain adaptation) shows the lowest overall performance, demonstrating that the combination of VEA, dual-backbone fusion, multi-task learning, and domain adaptation provides substantial synergistic benefits.

**TABLE 3:** Ablation study results.

Configuration	ACC			AUROC		
	Plus	Stage	Treatment	Plus	Stage	Treatment
VEA-net (ours)	85.44 ± 15.78	74.81 ± 10.17	56.80 ± 23.48	91.76 ± 4.16	88.26 ± 4.61	66.81 ± 15.87
W/o VEA	85.21 ± 16.71	73.46 ± 11.96	57.29 ± 22.59	91.55 ± 5.33	87.12 ± 4.96	63.07 ± 12.34
W/o dual-backbone	83.67 ± 15.11	75.37 ± 8.05	58.67 ± 25.64	88.15 ± 5.02	87.96 ± 3.69	62.84 ± 13.23
W/o task correlation	84.99 ± 15.96	75.01 ± 9.88	58.04 ± 24.22	89.08 ± 6.51	87.94 ± 3.07	62.45 ± 15.63
W/o domain adapt	85.26 ± 13.90	75.57 ± 9.73	54.94 ± 13.61	91.71 ± 4.09	87.87 ± 4.52	68.09 ± 10.71
*Domain Adaptation Variants*						
DANN-only (w/o MMD)	82.96 ± 8.21	67.87 ± 9.36	51.27 ± 7.71	86.47 ± 6.36	81.76 ± 9.94	60.27 ± 7.53
MMD-only (w/o DANN)	83.43 ± 7.84	73.27 ± 4.21	53.86 ± 2.35	88.27 ± 4.00	88.14 ± 3.51	60.36 ± 9.77
Baseline (ResNet50)	83.56 ± 19.34	72.28 ± 10.08	48.96 ± 16.99	88.47 ± 10.73	85.20 ± 5.61	62.95 ± 19.70

ACC and AUROC values are reported as mean ± standard deviation under 10-fold subject-level Group K-Fold across all three datasets for all ablation configurations, including the DANN-only and MMD-only variants.

### 4.4 Comparison study

We compare VEA-Net with several existing approaches for ROP analysis. Table 4 summarizes the comprehensive comparison. As shown in the table, VEA-Net achieves competitive performance across all three tasks compared to prior methods. Based on the 10-fold subject-level cross-validation results, we report the mean accuracy with standard deviation across folds: Plus detection accuracy 85.44% ± 15.78%, Stage classification accuracy 74.81% ± 10.17%, and Treatment decision accuracy 56.80% ± 23.48%. Because the compared methods may differ in implementation details and training dynamics, these comparisons are interpreted descriptively rather than as formal paired statistical tests. The methods compared in Table 4 are described as follows. SPIE (Graziani et al., 2019) is a convolutional approach for ROP classification that employs image enhancement preprocessing followed by a custom CNN architecture. VGG-ST (Luo et al., 2024) leverages a VGG-based backbone with a specialized staging module for multi-class ROP grading. ROPDeepX (Mohiy et al., 2026) uses a hybrid ResNet50–EfficientNet-B4 architecture that was shown to achieve strong performance on public ROP benchmarks. CdCL (Wei et al., 2023) introduces a contrastive learning-based framework that incorporates cross-dataset consistency to improve generalization across heterogeneous ROP data. Swin-Tiny is evaluated by us under the same protocol for direct comparison.

**TABLE 4:** Comparison with existing methods and backbone variants.

Method	Plus detection		Stage classification		Treatment decision	
	Acc	AUROC	Acc	AUROC	Acc	AUROC
SPIE Graziani et al. (2019)	81.14 ± 20.31	63.04 ± 11.63	65.82 ± 9.89	75.07 ± 5.14	44.77 ± 29.02	45.92 ± 16.91
VGG-ST Luo et al. (2024)	81.69 ± 20.35	68.10 ± 15.61	65.04 ± 9.81	72.30 ± 6.27	48.21 ± 26.60	48.78 ± 15.36
ROPDeepX Mohiy et al. (2026)	81.45 ± 20.35	62.87 ± 7.58	65.04 ± 11.56	74.06 ± 4.98	45.81 ± 25.90	48.86 ± 21.05
CdCL Wei et al. (2023)	81.57 ± 20.31	63.66 ± 11.08	64.18 ± 11.47	73.35 ± 6.61	44.22 ± 25.89	44.94 ± 15.60
Swin-tiny Liu Z. et al. (2021)	83.21 ± 16.41	90.00 ± 7.35	74.60 ± 9.17	87.43 ± 4.35	46.70 ± 22.94	65.52 ± 7.62
VEA-net (ours)	**85.44** ± **15.78**	**91.76** ± **4.16**	**74.81** ± **10.17**	**88.26** ± **4.61**	**56.80** ± **23.48**	**66.81** ± **15.87**

Test ACC and AUROC values are reported as mean ± standard deviation. Bold values indicate the best performance among all compared methods.

Compared to ROPDeepX, our model achieves higher Plus detection accuracy, which may be attributable to the VEA module’s enhanced vascular feature extraction capability. The multi-task CNN baseline shows lower performance, supporting the potential benefits of hierarchical consistency losses and domain adaptation. Overall, VEA-Net provides a unified three-task framework for Plus detection, stage classification, and auxiliary treatment decision support rather than isolated single-task predictions. The domain-adaptive ROP method shows competitive Plus detection but lacks multi-task capability and treatment decision support. Our dual-domain adaptation strategy provides superior cross-center generalization while maintaining task-specific performance, as evidenced by the consistent results across FARFUM, Ostrava, and A-Fundus test sets. To further validate our backbone selection, we also conducted an additional comparison using Swin-Tiny Transformer (Liu Z. et al., 2021) as a single-backbone baseline. The Swin-T model was configured with the same three-task classification heads, domain adaptation, and 10-fold subject-level cross-validation protocol, but without VEA modules or multi-branch fusion. As shown in the table, Swin-Tiny achieves comparable Plus detection and Stage classification, with estimated AUROC values of 90.0% ± 7.4%, 87.4% ± 4.4%, and 65.5% ± 7.6%. VEA-Net achieves higher mean treatment decision-making accuracy, suggesting the potential benefit of the dual-backbone design and vascular enhancement attention for capturing clinically informative features.

### 4.5 Per-center performance analysis

Table 5 reports per-center accuracy and AUROC. As shown in Table 5, performance varies substantially across centers. Ostrava achieves the highest Plus detection accuracy of 97.17% and Stage classification accuracy of 90.42%, while FARFUM-ROP shows lower performance with 59.83% on Plus detection and 50.19% on Stage classification. A-Fundus, lacking Plus disease and treatment labels, provides stage classification results of 66.15%. Several factors contribute to these differences. More specifically,the Ostrava’s Plus detection labels are heavily Normal-biased, meaning a trivial majority-class predictor would achieve approximately 89.5% accuracy. The observed 97.17% accuracy exceeds this baseline, indicating genuine discriminative ability, but the high accuracy is partly amplified by the skewed distribution. In contrast, FARFUM-ROP has a much more balanced distribution, making the classification task inherently more challenging and the accuracy figure more meaningful. In addition, the Ostrava provides only binary Plus disease labels, reducing the task to a simpler two-class problem. FARFUM-ROP provides three-class labels, requiring the model to discriminate the subtle Pre-Plus state, which is more nuanced and inherently more difficult. This label scheme difference means the Ostrava and FARFUM-ROP accuracy figures are not directly comparable. Moreover, the FARFUM-ROP serves as the primary source domain for supervised training (all three tasks are supervised here), while Ostrava functions primarily as a target domain in the domain adaptation framework. The high performance on Ostrava could partially reflect the model’s ability to leverage Ostrava’s large sample size during joint training with domain adaptation, combined with the relatively simple binary classification task described above.

**TABLE 5:** Per-center performance analysis.

Dataset	Plus detection		Stage classification		Treatment decision	
	Acc.	AUROC	Acc.	AUROC	Acc	AUROC
FARFUM-ROP	59.83 ± 23.25%	79.31 ± 19.02%	50.19 ± 22.33%	80.63 ± 17.19%	76.45 ± 17.19%	86.93 ± 10.93%
Ostrava	97.17 ± 3.74%	98.32 ± 0.72%	90.42 ± 7.47%	89.07 ± 5.75%	–	–
A-fundus	–	–	66.15 ± 7.17%	86.52 ± 5.17%	–	–

Ostrava and A-Fundus do not provide treatment annotations; A-Fundus does not provide Plus disease labels. Reported values reflect only samples with valid labels for each task.

### 4.6 Feature visualization

**FIGURE 3:**
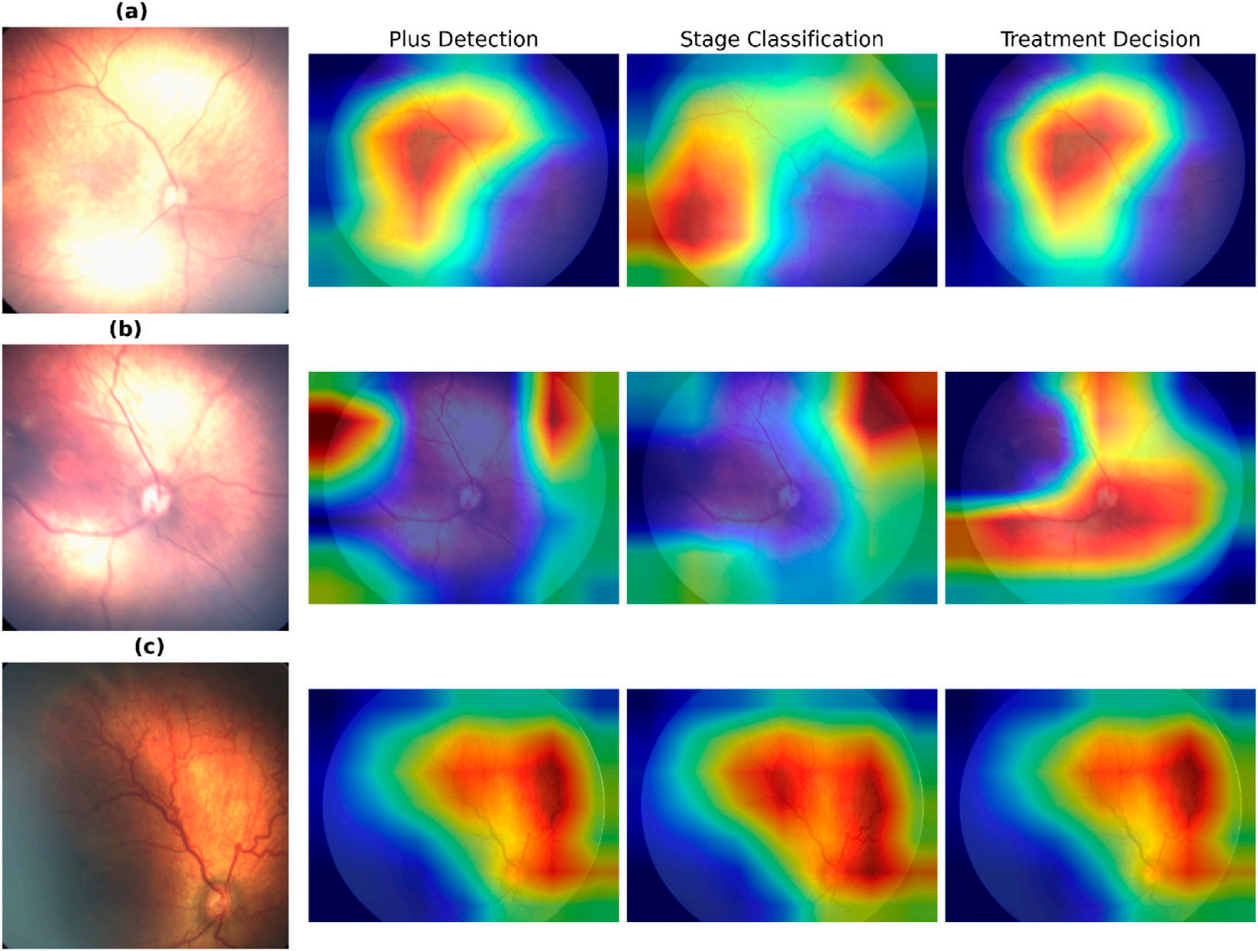
Grad-CAM visualizations for VEA-Net using the trained mode. Rows show representative fundus images with different Plus disease statuses (Normal, Pre-Plus, and Plus). Columns show the original image and Grad-CAM heatmaps for (1) Plus detection, (2) stage classification, and (3) treatment decision-making. The heatmaps reveal that Plus detection focuses on posterior pole vessels, stage classification extends attention to peripheral retinal regions, and treatment decision-making integrates both vascular activity and anatomical disease extent.

To identify which visual features contribute most to VEA-Net’s predictions, we employed Gradient-weighted Class Activation Mapping (Grad-CAM) to generate task-specific heatmaps. The visualizations were produced using the trained VEA-Net model. Figure 3 shows representative Grad-CAM heatmaps across three clinical tasks for images with different Plus disease statuses. For Plus detection, the model primarily focuses on the posterior pole and major retinal vessels, particularly regions showing vascular dilation, abnormal caliber, and tortuous trajectories. This attention pattern is consistent with the clinical definition of Plus disease, in which posterior pole vessel dilation and tortuosity are key diagnostic criteria. For stage classification, the attention maps extend beyond the posterior pole and include peripheral retinal regions where demarcation lines, ridges, neovascular proliferation, or tractional changes may appear. This broader spatial attention is consistent with the anatomical basis of ROP staging. For treatment decision-making, the heatmaps integrate information from both posterior pole vascular abnormalities and peripheral disease severity, reflecting the clinical process in which treatment recommendations depend on both disease activity and anatomical stage. The attention patterns are consistent with those reported in the previous version, confirming that the re-trained model maintains clinically meaningful visual focus.

## 5 Discussion

In this study, we proposed VEA-Net, a dual-backbone multi-task learning framework that integrates vascular enhancement attention, hierarchical multi-task learning, and domain adaptation for image-based ROP analysis. Evaluated on three public retrospective datasets comprising 8,636 retinal images from 737 unique subjects across Iran, the Czech Republic, and China. These results suggest that the proposed framework can learn useful representations across heterogeneous retrospective datasets under rigorous subject-level cross-validation, but they should not be interpreted as definitive evidence of prospective clinical deployment readiness.

The VEA module provides a targeted mechanism for capturing subtle vascular features that are clinically relevant to Plus disease. Our ablation study demonstrated that the most pronounced benefit of the VEA module is observed in the Treatment task, where removing it reduces Treatment AUROC by 3.74%, while Plus and Stage tasks remain relatively stable. This finding suggests the value of explicitly modeling vascular morphology, with particularly strong impact on treatment-relevant feature discrimination. Unlike conventional CNN architectures that treat retinal images as generic visual patterns (Brown et al., 2018; Chen et al., 2021), our VEA module employs multi-scale convolutions to capture vascular abnormalities at different spatial scales, directional selective filtering to enhance tortuous vessel patterns, and dual attention mechanisms to refine channel-wise and spatial feature representations. The Grad-CAM visualizations suggest that the VEA module tends to direct model attention to the posterior pole region where vascular dilation and tortuosity are clinically important, which is broadly consistent with established clinical criteria for Plus disease diagnosis (Chiang et al., 2021). Compared to previous approaches that either did not explicitly model subtle vascular features (Wu et al., 2022; Shoeibi et al., 2026) or used more complex spatial-frequency transformations (Cheng et al., 2025), our integrated VEA module achieves strong Plus detection accuracy of 85.44% without increasing annotation burden. These findings suggest that explicit vascular feature enhancement within the main classification pipeline can provide a useful inductive bias for ROP image analysis. The hierarchical multi-task learning architecture addresses a fundamental limitation in existing ROP research: the treatment of screening, staging, and treatment decision-making as isolated problems despite their inherent clinical interdependencies. Our framework jointly optimizes all three tasks while explicitly modeling their relationships through hierarchical consistency losses. The end-to-end learning approach enables the model to discover complex decision boundaries that may not be captured by isolated task learning or hand-crafted rules. The soft attention fusion mechanism further enhances this framework by dynamically adjusting the contribution of ResNet50 and EfficientNet-B4 based on input characteristics, learning to prioritize local vascular details for Plus detection while emphasizing global context for stage classification.

Although the three tasks are clinically related, multi-task learning may also introduce negative transfer when task-specific objectives are not fully aligned. In our setting, this risk is particularly relevant for the treatment decision task because treatment labels are available only in FARFUM-ROP and treatment recommendations may depend on clinical variables beyond fundus appearance. To mitigate potential task conflicts, VEA-Net uses task-specific prediction heads after the shared representation, allowing each task to preserve its own decision boundary. The differential weighting of the treatment loss increases its contribution during training relative to the other tasks, reducing the risk that the easier Plus detection task dominates representation learning. The hierarchical consistency loss further constrains the model toward clinically plausible combinations of Plus status, disease stage, and treatment recommendation. Nevertheless, we acknowledge that these mechanisms alleviate rather than eliminate negative transfer, and future work should include explicit gradient-conflict analysis or task-balancing strategies to further characterize inter-task optimization dynamics. Domain shift remains one of the most significant barriers to deploying medical AI models in real-world clinical settings (Guan and Liu, 2021). Our dual-domain adaptation strategy combines DANN with MMD to reduce center-specific feature bias across heterogeneous data sources. The ablation study showed that the MMD-only configuration yields Plus AUROC of 88.27%, Stage AUROC of 88.14%, and Treatment AUROC of 60.36%, while the DANN-only configuration yields Plus AUROC of 86.47%, Stage AUROC of 81.76%, and Treatment AUROC of 60.27%. The combined DANN + MMD configuration achieves the best per-task AUROC on all three tasks. These results confirm that DANN and MMD regularize complementary aspects of domain shift, and their combination provides consistent advantage across all three tasks.

Despite the promising results, several limitations should be acknowledged. First, the treatment decision task is supervised only by FARFUM-ROP because treatment annotations are unavailable in Ostrava and A-Fundus. Therefore, the generalizability of treatment recommendations to external populations cannot be considered fully validated. In real clinical practice, treatment decisions are influenced not only by fundus appearance but also by gestational age, birth weight, postmenstrual age, oxygen exposure, systemic condition, disease progression, and local clinical protocols (Chiang et al., 2021). The current treatment branch should therefore be regarded as an image-based auxiliary decision-support output rather than an autonomous treatment recommendation system. Second, the treatment decision task shows a larger standard deviation in cross-validation compared to Plus detection and stage classification. This instability is likely related to the smaller number of unique patients with treatment labels, class imbalance, and the inherently ambiguous boundary between close follow-up and active treatment. Third, although subject-level Group K-Fold splitting was applied to all three datasets, the number of unique subjects remains limited in some cohorts. This may increase the risk of overfitting to center-specific or patient-specific imaging patterns. Fourth, all datasets are retrospective public datasets. Although they represent different countries and imaging devices, they do not replace prospective validation in a completely unseen clinical center. Future studies should include prospective multi-center validation, external cohorts with treatment annotations, integration of clinical variables, uncertainty estimation, image-quality control, local calibration, per-class diagnostic metrics, and model compression for resource-constrained screening environments.

## 6 Conclusion

We presented VEA-Net, a unified DL framework for image-based ROP analysis that integrates vascular enhancement attention, dual-backbone feature extraction, hierarchical multi-task learning, and domain adaptation. Evaluated on three public retrospective datasets comprising 8,636 retinal images, VEA-Net achieved 85.44% ± 15.78% accuracy and 91.76% AUROC for Plus detection, 74.81% ± 10.17% accuracy and 88.26% AUROC for stage classification, and 56.80% ± 23.48% accuracy and 66.81% AUROC for auxiliary treatment decision support. The consistency between validation and test results suggests that the proposed vascular enhancement and domain adaptation strategies improve robustness across the evaluated public datasets. However, this finding should be interpreted as evidence of retrospective multi-dataset robustness rather than definitive proof of deployment readiness in unseen clinical centers. By integrating diagnostic assessment and auxiliary treatment decision support, VEA-Net represents a step toward AI-assisted ROP management. Future work will focus on prospective external validation, incorporation of patient-level clinical variables, uncertainty estimation, model compression, and evaluation in real-world screening workflows.

## Data Availability

The original contributions presented in the study are included in the article/supplementary material, further inquiries can be directed to the corresponding authors.
